# Deep learning-based computer-aided diagnosis in screening breast ultrasound to reduce false-positive diagnoses

**DOI:** 10.1038/s41598-020-79880-0

**Published:** 2021-01-11

**Authors:** Soo -Yeon Kim, Yunhee Choi, Eun -Kyung Kim, Boo-Kyung Han, Jung Hyun Yoon, Ji Soo Choi, Jung Min Chang

**Affiliations:** 1 Department of Radiology, Seoul National University Hospital, Seoul National University College of Medicine, 101 Daehak-ro, Jongno-gu, Seoul, 03080 Republic of Korea; 2 Medical Research Collaborating Center, Seoul National University Hospital, Seoul, Republic of Korea; 3 Department of Radiology and Research Institute of Radiological Science, Severance Hospital, Yonsei University College of Medicine, Seoul, Republic of Korea; 4 Department of Radiology and Center for Imaging Science, Samsung Medical Center, Sungkyunkwan University School of Medicine, Seoul, Republic of Korea

**Keywords:** Imaging, Ultrasound, Cancer, Breast cancer, Biological techniques, Software, Computational biology and bioinformatics, Machine learning, Software

## Abstract

A major limitation of screening breast ultrasound (US) is a substantial number of false-positive biopsy. This study aimed to develop a deep learning-based computer-aided diagnosis (DL-CAD)-based diagnostic model to improve the differential diagnosis of screening US-detected breast masses and reduce false-positive diagnoses. In this multicenter retrospective study, a diagnostic model was developed based on US images combined with information obtained from the DL-CAD software for patients with breast masses detected using screening US; the data were obtained from two hospitals (development set: 299 imaging studies in 2015). Quantitative morphologic features were obtained from the DL-CAD software, and the clinical findings were collected. Multivariable logistic regression analysis was performed to establish a DL-CAD-based nomogram, and the model was externally validated using data collected from 164 imaging studies conducted between 2018 and 2019 at another hospital. Among the quantitative morphologic features extracted from DL-CAD, a higher irregular shape score (*P* = .018) and lower parallel orientation score (*P* = .007) were associated with malignancy. The nomogram incorporating the DL-CAD-based quantitative features, radiologists’ Breast Imaging Reporting and Data Systems (BI-RADS) final assessment (*P* = .014), and patient age (*P* < .001) exhibited good discrimination in both the development and validation cohorts (area under the receiver operating characteristic curve, 0.89 and 0.87). Compared with the radiologists’ BI-RADS final assessment, the DL-CAD-based nomogram lowered the false-positive rate (68% vs. 31%, *P* < .001 in the development cohort; 97% vs. 45% *P* < .001 in the validation cohort) without affecting the sensitivity (98% vs. 93%, *P* = .317 in the development cohort; each 100% in the validation cohort). In conclusion, the proposed model showed good performance for differentiating screening US-detected breast masses, thus demonstrating a potential to reduce unnecessary biopsies.

## Introduction

Mammography is an established modality for the early detection of breast cancer and reduction of breast cancer-related mortality and morbidity^1^. However, as mammography alone shows a low sensitivity in women with dense breasts, various supplementary imaging modalities have been investigated for screening breast cancer^2^. Supplemental screening breast ultrasound (US) is a widely used adjunctive tool that allows the detection of additional breast cancers in women with negative mammography^2^. Although the number of cancers detected varies with the prevalence of cancer, it is generally modest, with an average of 3–4 cancers per 1000 US examinations^3–7^. As most cancers detected using screening US are small, invasive and node-negative, early-stage cancer detection and management may confer a mortality benefit^3–7^.

Despite the well-known benefits of screening breast US, a major limitation is presented by a substantial increase in false-positive findings with low specificities and low positive predictive values^8^. The Breast Imaging Reporting and Data System (BI-RADS) lexicon for breast US has contributed to the standardization of lesion characterization, description, and reporting; moreover, it has proven effective in differentiating benign and malignant breast lesions^9–12^. However, efforts to detect more cancers using screening US have led to excessive recalls and false positive biopsies. In addition, cancers detected using screening US tend to be small in size, without calcifications, and lack typical malignant features compared with palpable breast cancers or breast cancers detected using screening mammography^13,14^. Hence, the task of classifying breast masses detected using screening US as benign or malignant is challenging. Moreover, the assessment of masses detected using screening US entails concerns regarding operator dependency.

Artificial intelligence, particularly involving deep learning algorithms, is gaining extensive attention owing to its excellent performance in image recognition tasks^15^. A deep learning based computer-aided diagnosis (DL-CAD) software has been recently developed and employed for breast mass differentiation in clinical practice^16–21^. Reports on the use of the dichotomized final assessments (“possibly benign” or “possibly malignant”) rendered by the current commercial DL-CAD software in patients with variable conditions showed the potential of DL-CAD with regard to improving the diagnostic accuracy and specificity^16–21^. However, to the best of our knowledge, no studies have focused explicitly on DL-CAD performance considering selected patients with breast masses detected using screening US. Considering the small size and less obvious morphologic characteristics of breast masses detected using screening US, the quantitative information derived from the DL-CAD software could provide additional details missed by the expert radiologists; furthermore, such information could prove to be substantially useful for the differentiation of breast masses while facilitating patient management in the clinical setting.

Therefore, the purpose of this study was to develop a diagnostic model that incorporates the quantitative morphologic features extracted from DL-CAD to improve the differential diagnosis of breast masses detected using screening US and reduce false-positive diagnoses.

## Results

### Characteristics of the development and validation cohorts

The quantitative morphologic scores were obtained using the DL-CAD software for the development and validation sets (463 patients, mean age, 45 years; range, 19–81). Surgery was performed on all lesions with malignant biopsy results (n = 52) and some of the lesions with benign biopsy results (n = 81). Clinical and imaging follow-up was performed using US (mean follow-up time, 12 months; range, 6–23 months) for the non-excised lesions proven to be benign through biopsy (n = 330), and lesion stability was confirmed in all the cases. The characteristics of the development and validation cohorts are summarized in Table 1. The age of the patients and size of the breast masses in the US were comparable between the two cohorts (*P* = 0.937 and 0.444, respectively). The proportion of BI-RADS category 3 lesions was significantly higher in the development cohort than in the validation cohort (28.1% [84 of 299] vs. 2.4% [4 of 164], *P* < 0.001). In the development cohort, there were 256 (85.6%) benign and 43 (14.4%) malignant (33 invasive ductal carcinomas, 7 ductal carcinomas in situ, 1 invasive lobular carcinoma, 1 mucinous carcinoma, and 1 adenoid cystic carcinoma) masses. In the validation cohort, there were 155 (94.5%) benign and 9 (5.5%) malignant (7 invasive ductal carcinomas and 2 ductal carcinomas in situ) masses. The proportion of malignancy was significantly higher in the development cohort than in the validation cohort (14.4% vs. 5.5%, *P* = 0.004). Among the quantitative morphologic scores extracted from the DL-CAD software (Supplementary Table S1), the round shape score and all descriptor scores of the echo pattern, margin, and posterior features were significantly different between the two cohorts (all *P* < 0.001). However, the oval shape score, irregular shape score, and parallel and non-parallel orientation scores were all comparable between the two cohorts (*P* = 0.545–0.859).

**Table 1:** Characteristics of the development and validation cohorts.

Characteristic	Development cohort (n = 299)	Validation cohort (n = 164)	*P* value
Age (y)	45 ± 12 (19–81)	45 ± 10 (23–74)	.937
Size on US (cm)	1.1 ± 0.6 (0.4–3.2)	1.1 ± 0.5 (0.3–3.4)	.444
**Radiologist’s BI-RADS assessment**			
3	84 (28.1)	4 (2.4)	< .001
4A	174 (58.2)	146 (89.0)	
4B	20 (6.7)	11 (6.7)	
4C	14 (4.7)	3 (1.8)	
5	7 (2.3)	0 (0)	
**Pathology**			
Benign	256 (85.6)	155 (94.5)	.004
Malignant	43 (14.4)	9 (5.5)	

BI-RADS, Breast Imaging Reporting and Data System. Data for the age and size are expressed in terms of the mean ± standard deviation (ranges). Data for the BI-RADS final assessment and pathology are presented as numbers (percentages).

### Characteristics associated with malignant masses in the development cohort

The characteristics of benign (n = 256) and malignant (n = 43) breast lesions in the development cohort were compared using univariable logistic regression analysis, as presented in Table 2. Women with malignant breast lesions were older than those with benign lesions (mean age, 54 vs. 44 years, *P* < 0.001). All malignant breast lesions except one (97.7%, 42 of 43) had a final assessment of BI-RADS 4A or higher. In contrast, 67.6% (173 of 256) of the benign breast lesions had a final assessment of BI-RADS 4A or higher (*P* = 0.003). With regard to the differences in the quantitative morphologic scores obtained from the DL-CAD software between the benign and malignant breast lesions, the malignant breast lesions had a lower oval shape score (median, 0.15 vs. 0.77, *P* < 0.001), higher irregular shape score (0.82 vs. 0.12, *P* < 0.001), lower parallel orientation score (0.72 vs. 0.99, *P* < 0.001), higher non-parallel orientation score (0.28 vs. 0.01, *P* < 0.001), lower circumscribed margin score (0.11 vs. 0.98, *P* < 0.001), higher spiculated margin score (0 [interquartile ranges, 0–0.01] vs. 0 [0–0], *P* = 0.037), higher microlobulated margin score (0.16 vs. 0.01, *P* < 0.001), lower posterior enhancement score (0.14 vs. 0.38, *P* < 0.001), and higher posterior shadowing score (0.01 vs. 0, *P* = 0.011), compared with the benign breast lesions. In the multivariable analysis, four variables were independently associated with malignancy (Table 3): BI-RADS 4A or higher (odds ratio [OR], 14.7 [95% confidence interval (CI) 1.7, 125.6], *P* = 0.014), older age (OR, 1.1 [95% CI 1.0, 1.1], *P* < 0.001), higher irregular shape score (OR, 5.1 [95% CI 1.3, 19.4], *P* = 0.018), and lower parallel orientation score (OR, 0.1 [95% CI 0, 0.6], *P* = 0.007) obtained from the DL-CAD software.

**Table 2:** Characteristics of the benign and malignant breast lesions in the development cohort.

Characteristic	Benign (n = 256)	Malignant (n = 43)	Univariable odds ratio (95% CI)	Univariable *P* value
Age (y)	44 ± 11 (19–78)	54 ± 13 (22–81)	1.1 (1.0, 1.1)	< .001
Size on US (cm)	1.1 ± 0.5 (0.4–3.2)	1.3 ± 0.9 (0.4–3.2)	1.5 (0.9, 2.4)	.124
*Radiologist’s BI-RADS assessment*				
3	83 (32.4)	1 (2.3)	Reference	
≥ 4A	173 (67.6)	42 (97.7)	20.2 (2.7, 148.9)	.003
**Quantitative morphology scores from the DL-CAD software**				
*Characteristic*				
Descriptor				
*Shape*				
Round	0.01 (0, 0.05)	0.01 (0, 0.08)	0.6 (0, 11.3)	.734
Oval	0.77 (0.36, 0.93)	0.15 (0.05, 0.51)	0.04 (0.01, 0.14)	< .001
Irregular	0.12 (0.03, 0.49)	0.82 (0.44, 0.92)	18.6 (6.3, 54.8)	< .001
*Orientation*				
Parallel	0.99 (0.95, 0.99)	0.72 (0.24, 0.96)	0.03 (0.01, 0.1)	< .001
Not parallel	0.01 (0, 0.05)	0.28 (0.05, 0.76)	30.9 (9.4, 101.0)	< .001
*Margin*				
Circumscribed	0.98 (0.41, 0.99)	0.11 (0, 0.84)	0.2 (0.1, 0.4)	< .001
Indistinct	0 (0, 0.05)	0.02 (0, 0.46)	2.3 (0.8, 6.6)	.107
Spiculated	0 (0, 0)	0 (0, 0.01)	9.6 (1.2, 80.9)	.037
Angular	0 (0, 0)	0 (0, 0)	1.2 (0.04, 38.9)	.902
Microlobulated	0.01 (0, 0.07)	0.16 (0.01, 0.72)	6.5 (2.4, 17.5)	< .001
*Posterior features*				
No	0.44 (0.18, 0.71)	0.58 (0.20, 0.78)	2.1 (0.7, 6.3)	.195
Enhancement	0.38 (0.12, 0.72)	0.14 (0.01, 0.30)	0.1 (0.03, 0.4)	< .001
Shadowing	0 (0, 0.01)	0.01 (0, 0.32)	4.1 (1.4, 12.3)	.011
Combined	0 (0, 0)	0 (0, 0.02)	2.0 (0.3, 13.4)	.465
*Echo pattern*				
Anechoic	0 (0, 0)	0 (0, 0)	1.1 (0.9, 1.2)	.281
Hyperechoic	0 (0, 0)	0 (0, 0)	3.9 (0, 836.3)	.618
Complex	0 (0, 0)	0 (0, 0)	0.4 (0, 37.2)	.671
Hypoechoic	0.84 (0.19, 0.98)	0.91 (0.28, 0.99)	1.7 (0.7, 4.2)	.292
Isoechoic	0.05 (0, 0.46)	0.01 (0, 0.29)	0.6 (0.2, 1.7)	.312
Heterogeneous	0 (0, 0)	0 (0, 0.03)	2.7 (0.6, 12.9)	.213

BI-RADS, Breast Imaging Reporting and Data System, CI, confidence interval, DL-CAD, deep learning-based computer-aided diagnosis.

Data for the age and size are expressed in terms of the mean ± standard deviation (ranges). Data for the BI-RADS final assessment are presented as numbers (percentages). Data for quantitative morphologic scores from the DL-CAD software are presented as median (25th percentile, 75th percentile).

**Table 3:** Multivariable logistic regression analysis for predicting malignancy.

	Odds ratio (95% CI)	*P* value
Age	1.1 (1.0, 1.1)	< .001
Radiologist’s BI-RADS assessment ≥ 4A	14.7 (1.7, 125.6)	.014
Irregular shape score	5.1 (1.3, 19.4)	.018
Parallel orientation score	0.1 (0, 0.6)	.007

BI-RADS, Breast Imaging Reporting and Data System; CI, confidence interval.

### Development and validation of a nomogram

**Figure 1:**
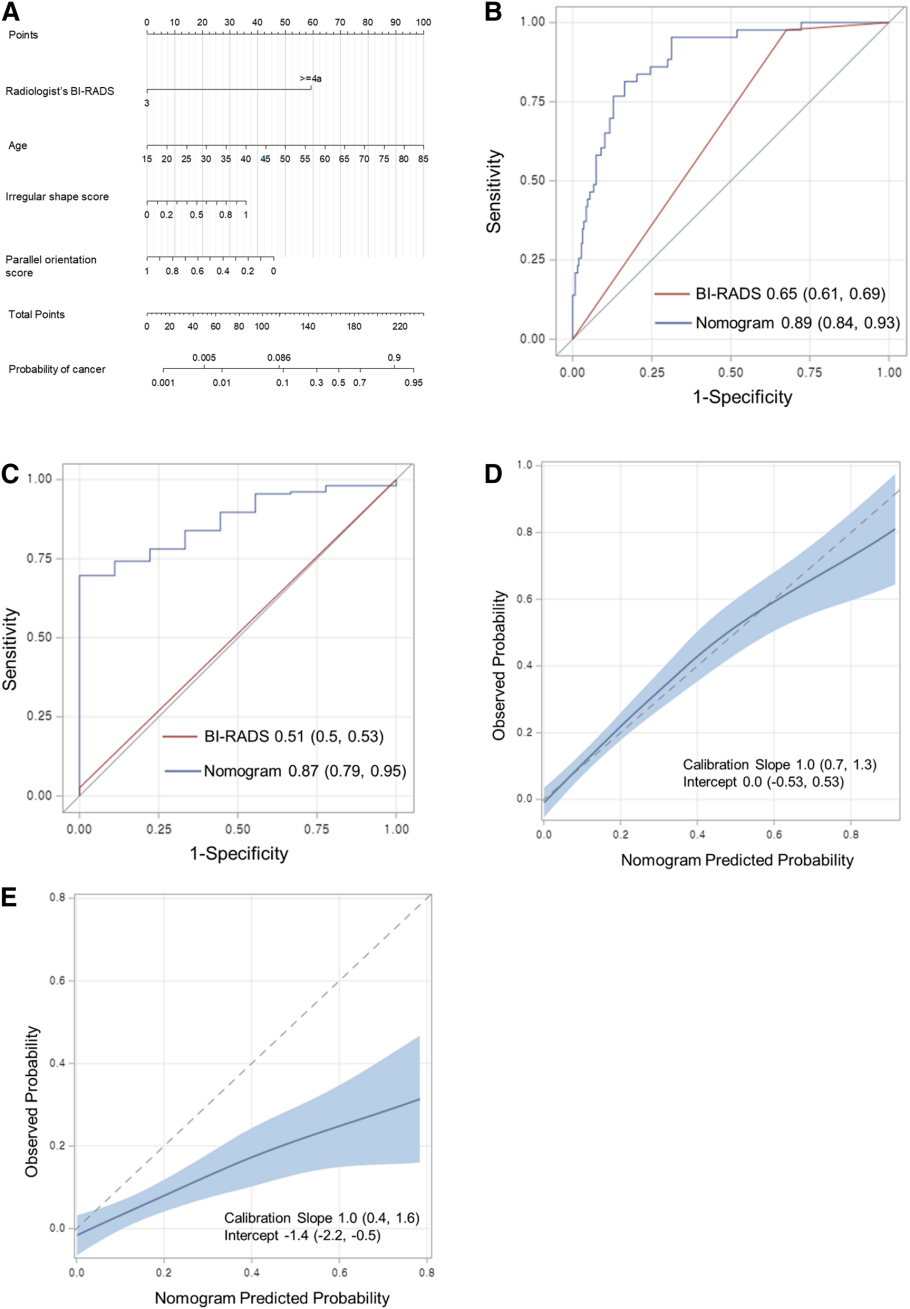
Nomogram to predict the malignancy in screening US-detected breast masses with receiver operating characteristic (ROC) curves and calibration plots. (**a**) Nomogram incorporating quantitative morphologic scores of shape and orientation from the deep learning based computer-aided diagnosis, age, and Breast Imaging Reporting and Data System (BI-RADS) final assessment category as variables was developed. Each point that corresponds to each variable is on the uppermost point scale. The sum of each point is the total point. The total points projected at the bottom scale indicate the probability of breast cancer. The ROC curves are compared between the nomogram and the radiologist’s BI-RADS final assessment in the (**b**) development cohort and (**c**) validation cohort. Numbers in the curves are area under the ROC curve (AUC) with 95% confidence intervals in the parentheses. The calibration plots of the nomogram are presented in the (**d**) development cohort and (**e**) validation cohort. Numbers in the parentheses are 95% confidence intervals.

A diagnostic model incorporating the four aforementioned variables was developed and presented as a nomogram (Fig. 1a). In the development cohort, the AUC of the radiologists’ BI-RADS final assessment was 0.65 (95% CI 0.61, 0.69), whereas the AUC in the case of the diagnostic model was 0.89 (95% CI 0.84, 0.93) (Fig. 1b). The bootstrap-corrected AUC of the diagnostic model was 0.87. In the validation cohort, the diagnostic model yielded a higher AUC than the radiologists’ BI-RADS assessment (0.87 [95% CI 0.79, 0.95] vs. 0.51 [95% CI 0.50, 0.53], *P* < 0.001) (Fig. 1c). In the development cohort, the calibration plot showed good agreement between the predicted and observed probabilities (Fig. 1d); the calibration slope and intercept were close to 1 (95% CI 0.7, 1.3) and 0 (95% CI − 0.5, 0.5), respectively. The bootstrap-corrected estimates for the calibration were 0.84 and − 0.75 for the slope and intercept, respectively. However, in the validation cohort, the observed probability was lower than the predicted probability (Fig. 1e) with a calibration slope of 1 (95% CI 0.4, 1.6) and calibration intercept of − 1.4 (95% CI − 2.2, − 0.5).

### Application of the nomogram to reduce unnecessary biopsies

**Figure 2:**
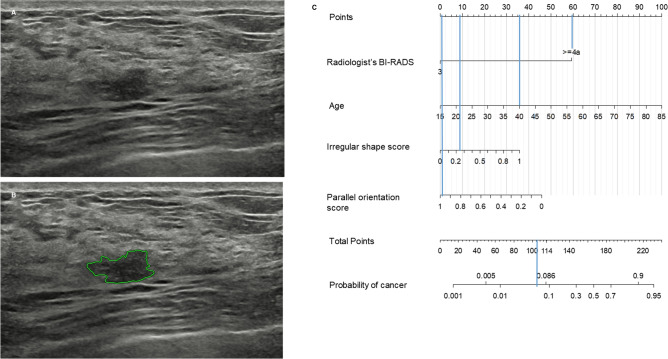
Benign breast mass correctly classified by the nomogram. (**a**) A 40-year-old woman in the validation cohort had a 1.0-cm breast mass detected by screening US. This mass assessed as Breast Imaging Reporting and Data System (BI-RADS) 4A by a radiologist was subject to US-guided 14-gauge core needle biopsy. The biopsy result was fibrocystic change and the mass was stable on 12 months follow-up US. (**b**) The deep learning based computer-aided diagnosis software assigned an irregular shape score of 0.25 and parallel orientation score of 0.98. (**c**) This mass has 105 points on the nomogram, which was lower than the cut-off of 114. A false-positive biopsy could be omitted safely if the nomogram was applied.

**Figure 3:**
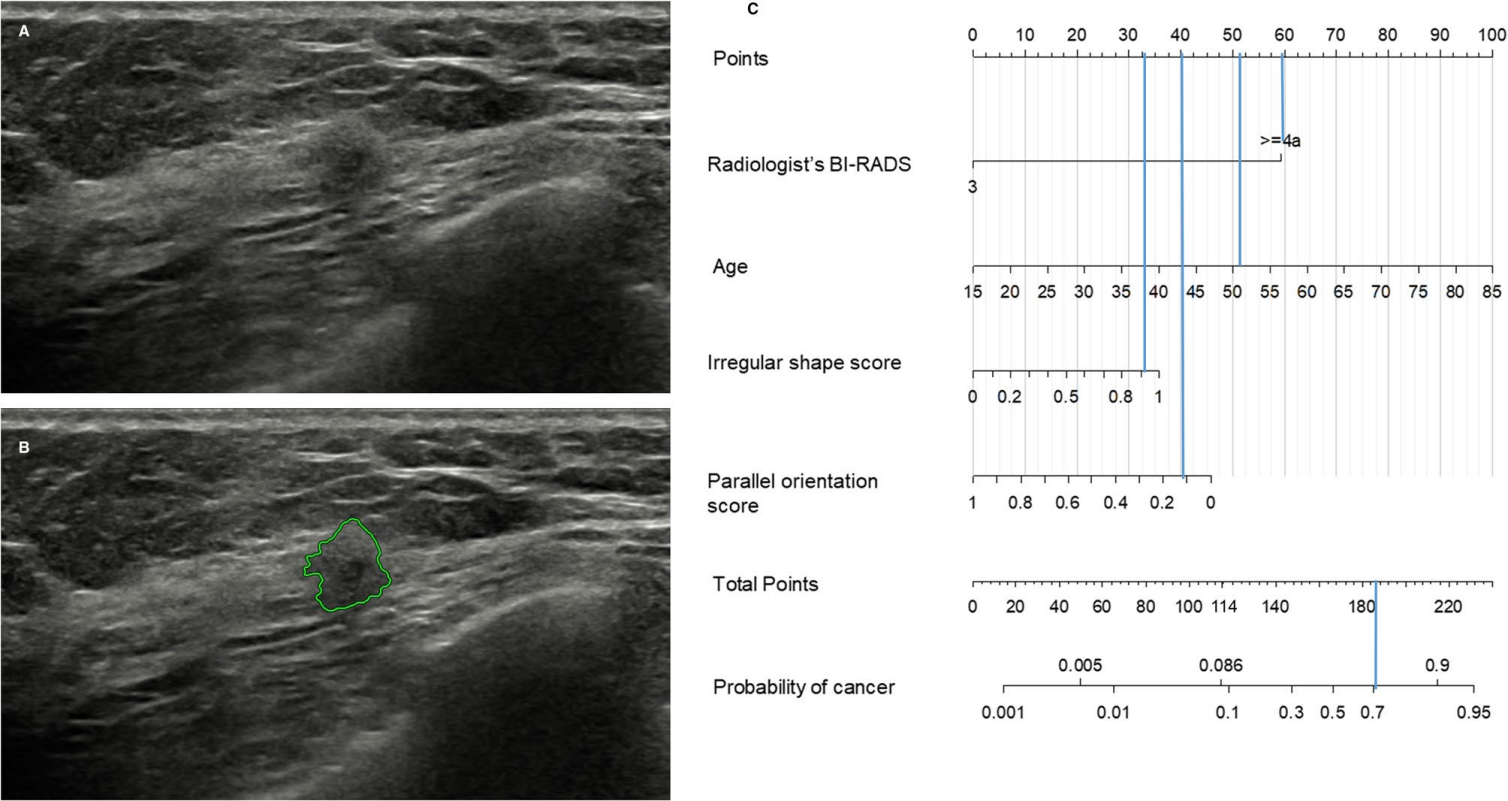
Malignant breast mass correctly classified by the nomogram. (**a**) A 51-year-old woman in the validation cohort had a screening US-detected 0.9-cm breast mass assigned as Breast Imaging Reporting and Data System (BI-RADS) 4A. The mass was confirmed as grade 1 invasive ductal carcinoma (pT1N0M0, estrogen receptor positive, progesterone receptor negative, human epidermal growth factor receptor type 2 negative) by US-guided 14-gauge core needle biopsy and subsequent surgery. (**b**) The deep learning based computer-aided diagnosis (DL-CAD) software assigned irregular shape score of 0.90 and parallel orientation score of 0.12. (**c**) This mass has 185 points on the nomogram, which was higher than the cut-off of 114, requiring biopsy. The nomogram incorporating quantitative morphologic scores from DL-CAD software correctly identified the true cancer.

After obtaining the risk scores from the diagnostic model, the optimal cut-off of the nomogram was determined to be 114 points to maximally improve the specificity with maintaining the sensitivity to 95% or higher. A comparison of the false positive rates, biopsy rates, and sensitivities between the BI-RADS final assessment and the diagnostic model using the nomogram with the determined cut-off is presented in Table 4. On application of the diagnostic model, the false positive rate significantly decreased in both the development cohort (68% [95% CI 62, 73; 173 of 256] vs. 31% [95% CI 26, 37; 80 of 256], *P* < 0.001) and validation cohort (97% [95% CI 95, 99; 151 of 155] vs. 45% [95% CI 37, 52; 69 of 155], *P* < 0.001). Moreover, the biopsy rate significantly decreased in both the development cohort (72% [95% CI 67, 77; 215 of 299] vs. 41% [95% CI 35, 46; 121 of 299], *P* < 0.001) and validation cohort (98% [95% CI 96, 100; 160 of 164] vs. 48% [95% CI 40, 55; 78 of 164], *P* < 0.001). The sensitivity did not significantly decrease in both the development cohort 98% [95% CI 88, 100; 42 of 43] vs. (95% [95% CI 84, 99; 41 of 43], *P* = 0.317) and validation cohort (100% [95% CI 66%, 100%; 9 of 9] vs. 100% [95% CI 66%, 100%; 9 of 9], *P* = not applicable). When the nomogram threshold was applied to breast masses with BI-RADS 4A, 88 (50%) of the 174 masses in the development cohort and 78 (53%) of the 146 masses in the validation cohort were correctly reclassified as benign without missing any cancers (Figs. 2 and 3).

**Table 4:** Comparison of the false positive rate, biopsy rate, and sensitivity between the radiologist’s BI-RADS assessment and the proposed nomogram.

	False positive rate		Biopsy rate		Sensitivity	
	Development cohort	Validation cohort	Development cohort	Validation cohort	Development cohort	Validation cohort
Radiologist’s BI-RADS ≥ 4A	68 (62, 73) [173/256]	97 (95, 99) [151/155]	72 (67, 77) [215/299]	98 (96, 100) [160/164]	98 (88, 100) [42/43]	100 (66, 100) [9/9]
Nomogram ≥ 114	31 (26, 37) [80/256]	45 (37, 52) [69/155]	41 (35, 46) [121/299]	48 (40, 55) [78/164]	95 (84, 99) [41/43]	100 (66, 100) [9/9]
*P* value	< .001	< .001	< .001	< .001	.317	NA

Data in parentheses are the 95% confidence intervals, and the data in brackets are the numerators/denominators.

BI-RADS, Breast Imaging Reporting and Data System.

## Discussion

In this study, the DL-CAD based nomogram for the differential diagnosis of breast masses detected by screening US was developed and validated using the multicenter cohorts. Among the quantitative morphologic features obtained from the DL-CAD software, ‘shape’ and ‘orientation’ information were the most beneficial features in predicting malignancy. In comparison with the radiologists’ BI-RADS final assessment alone, our nomogram incorporating the DL-CAD based quantitative information, patient age, and the BI-RADS assessments, improved AUCs and lowered the false-positive rates without decreasing the sensitivities in the differential diagnosis of breast masses detected by screening US.

To date, various studies have attempted to overcome the limitations of screening breast US, including a low positive predictive value and increase in false-positive biopsies^22^. The use of elastography or color Doppler US, in addition to grayscale US images, has been suggested and used in clinical practice^23^. However, in these techniques, the additional time required for image acquisition and operator dependency with regard to both image acquisition and interpretation remain as limitations^24^. In this respect, the use of DL-CAD with grayscale US images could provide objective, quantitative information, enabling the proposed method to serve as a second reader assisting radiologists to effectively reduce unnecessary biopsies and increase cost-effectiveness. The detection of subtle differences in the mass features among grayscale US images is promising, as this can also be applied to an automated breast US system.

With regard to creating the diagnostic model, the development and validation cohorts did not share common mass features and ratios of malignancy; however, good discrimination performance was noted, implying high compatibility with a slightly different cohort. The use of the developed nomogram lead to improved AUC values and lower false positive rates without reducing the sensitivity, in contrast with using only BI-RADS; thus, this is an effective method for reducing the false-positive biopsies associated with supplemental US exams. These results are concordant with those of previous studies that consistently reported the utility of DL-CAD software for improving the diagnostic specificity of both experienced and less-experienced radiologists^16–21^. However, in previous studies, their performances were evaluated on a single study population with a given dichotomized output from the DL-CAD software. In this study, the individual quantitative data of the DL-CAD software were explored for the first time.

Among the morphologic features of the grayscale US images assessed with the DL-CAD software, the shape and orientation were the most reliable US features for characterizing the screening US-detected breast masses. In contrast, the echogenicity and posterior features were the least reliable. The results of this study are consistent with those of prior studies by Hong et al.^25^ and Elverici et al.^26^, which investigated the US features useful in diagnosing non-palpable breast lesions. An irregular shape, spiculated margin, and non-parallel orientation are well-known features highly predictive of malignancy^25,26^. An irregular shape might reflect the inconsistent growth and advancement of the lesion edge, whereas the non-parallel orientation feature might suggest the spread of the lesion through the tissue-plane boundaries, both of which are substantially indicative of a malignant process^25^. Margin assessment is somewhat challenging for small-sized lesions despite the use of high-spatial-resolution US equipment^9^. In concordance with the study conducted by Elverici et al.^26^, the margin was not independently associated with malignancy in the screening-US-detected masses evaluated in this study. In the study performed by Chen et al.^27^, the reliable US features differed according to the size of the breast masses; furthermore, for breast masses ≤ 1 cm in diameter, an irregular shape and not smooth margin were associated with malignancy. The reliable US features predictive of malignancy might differ depending on the size and palpability of the breast masses.

This study has several limitations. First, this was a retrospective study, and the results were dependent on the composition of the data, whose size was limited. Owing to the small number of malignant lesions and the low proportion of BI-RADS category 3 masses in the validation cohort, a tendency to overestimate was noted^28^. Further improvements must be achieved via larger and prospective studies before actual clinical use. Second, although BI-RADS 3 masses that were biopsied on the request of a clinician or patient were included in this study, the inclusion of BI-RADS 3 masses may not reflect the usual population requiring biopsies. Third, we only applied our DL-CAD system for women who were scheduled for biopsy. In our study, we do not have data on entire screening cohort, and the patients who were not performing biopsy for their mass were not included. Fourth, only one representative US image was used for each case; thus, variability could still exist in lesion assessments performed by the DL-CAD software and radiologists. In addition, we used the quantitative scores extracted from the DL-CAD software, which is not displayed automatically in current clinical version. In the future, providing the quantitative information as an output should be considered based on our results. The modified software would improve the diagnostic accuracy even in challenging situations, such as in the case of breast cancers detected using screening US. Lastly, the risk status of the patients, such as a family history of breast cancer and BRCA mutation information, were not considered.

In conclusion, the proposed DL-CAD-based diagnostic model showed good performance with regard to the lesion differentiation of breast masses detected using screening US. The quantitative DL-CAD information can provide a more accurate diagnosis and potentially lead to a decrease in false positive biopsies without overlooking cancers.

## Methods

Samsung Medison Co. (Seongnam, Korea) provided technical support with regard to analyzing the US images using the DL-CAD software (S-Detect; Samsung Medison Co., Seongnam, Korea) and obtaining outputs from the software. However, Samsung Medison Co. was not involved in the study design, data collection, statistical analysis, data interpretation, or manuscript preparation. The institutional review boards of Seoul National University Hospital (No. 1901-110-1005), Severance Hospital (4-2019-0112), and Samsung Medical Center (2019-02-051-001) approved this multicenter retrospective study, and the requirement for written informed consent was waived for re-analyzing data from prospective cohort study. All research was performed in accordance with relevant guidelines and regulations.

### Patients

**Figure 4:**
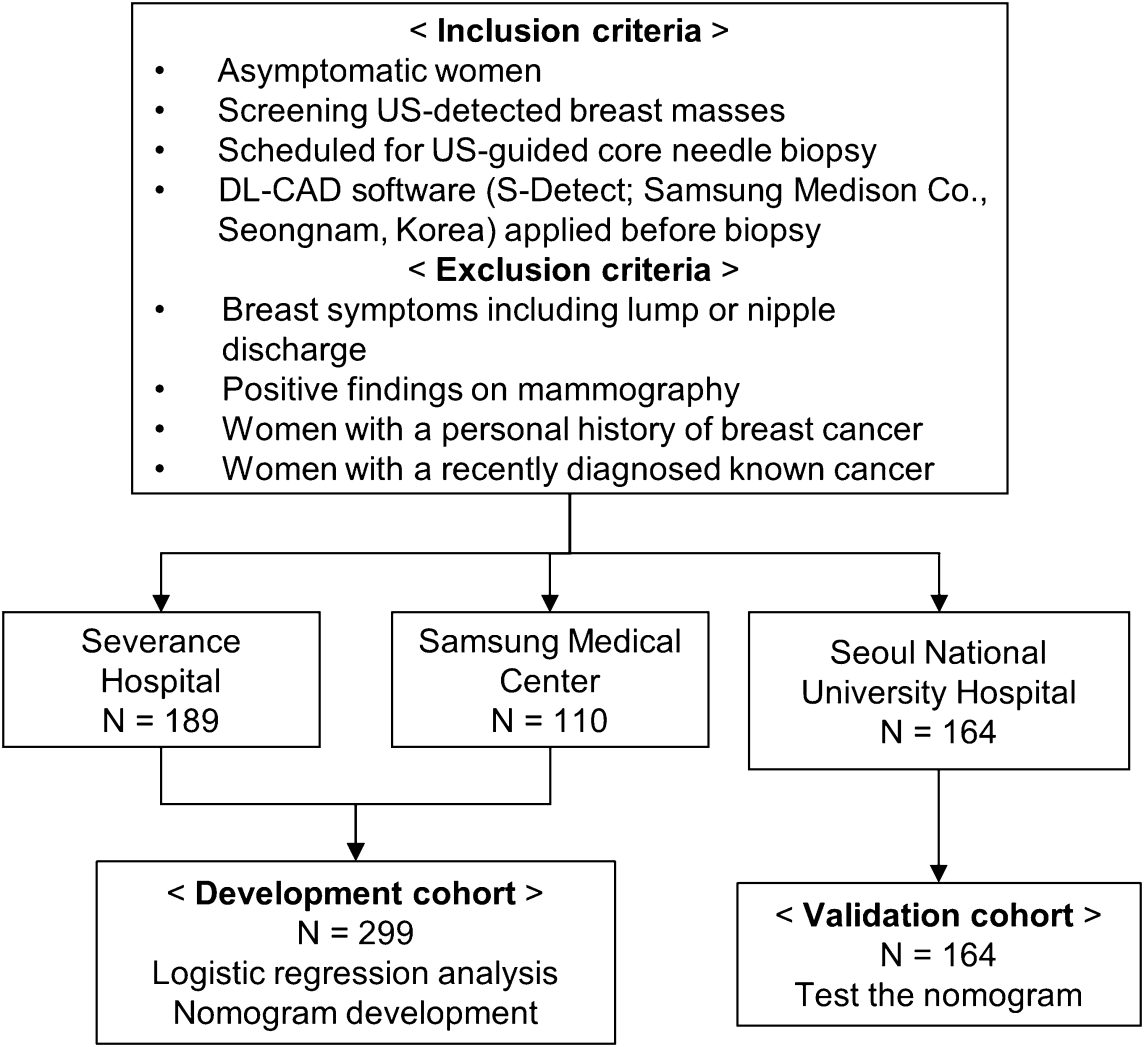
Flowchart of patient selection. DL-CAD = deep learning based computer-aided diagnosis.

With regard to the development cohort, the US images were collected from the prospective cohort studies performed at Severance Hospital and Samsung Medical Center, Seoul, Korea from January to December 2015. From this database, we identified patients according to the following inclusion and exclusion criteria. The inclusion criteria were asymptomatic women with breast masses detected using screening US, were scheduled for US-guided core needle biopsy, and the DL-CAD software was applied to the breast masses before performing US-guided core needle biopsy in all of the included cases. The exclusion criteria were women with breast symptoms, including lump or nipple discharge, with positive findings (BI-RADS final assessment category 0, 3, 4, or 5) obtained via mammography; the exclusion criteria also included a personal history of breast cancer, with recently diagnosed, known cancer. In total, 299 women who fulfilled the aforementioned inclusion criteria comprised the development set (mean age, 45 years; range, 19–81 years). To form an independent external validation cohort, we recruited patients using the same inclusion and exclusion criteria as those for the development cohort in Seoul National University Hospital, Seoul, Korea between February 2018 and June 2019. A total of 164 women (mean age, 45 years; range, 23–74 years) comprised the validation cohort (Fig. 4).

### DL-CAD application

All masses included in this study were scheduled to undergo US-guided biopsy according to the predetermined BI-RADS category. The BI-RADS category was determined solely based on grayscale US images without DL-CAD information. As per the institutional rules for biopsy, all BI-RADS category 4 or 5 masses undergo biopsy, and upon the request of a clinician or patient, BI-RADS category 3 masses can be scheduled for biopsy. On the day of the scheduled biopsy, the DL-CAD software was applied using 5–12 MHz linear probes and a real-time US system (RS 80A with Prestige and RS85A Prestige; Samsung Medison Co., Seongnam, Korea) before biopsy was performed by six dedicated breast radiologists (J.H.Y. and E.-K.K. in Severance Hospital, J.S.C. and B.-K.H. in Samsung Medical Center, and S.-Y.K. and J.M.C. in Seoul National University Hospital) with 6–23 years of experience. During the DL-CAD software operation, the radiologists first selected a single representative image for each mass. The representative image included the area with the most suspicious US features. The radiologists indicated the center of the mass on the selected image, and then a region of interest (ROI) was automatically drawn along the border of the mass. Manual corrections were made by the radiologists if the mass boundary was inadequately drawn by the DL-CAD software. The ROI-based US features were automatically analyzed according to the 5th edition of the BI-RADS lexicon^11^: shape (round, oval, and irregular), orientation (parallel and not parallel), margin (circumscribed, indistinct, spiculated, angular, and microlobulated), posterior feature (no posterior feature, posterior enhancement, posterior shadowing, and combined), echo pattern (anechoic, hyperechoic, complex, hypoechoic, isoechoic, and heterogeneous). The final assessments were provided in the dichotomized form as “possibly benign” and “possibly malignant.” The decisions regarding performing US-guided biopsy and the predetermined BI-RADS category were not altered based on the DL-CAD software results. Immediately after acquiring the DL-CAD images, the scheduled biopsy was performed.

### Extraction of quantitative morphologic scores from the DL-CAD software

**Figure 5:**
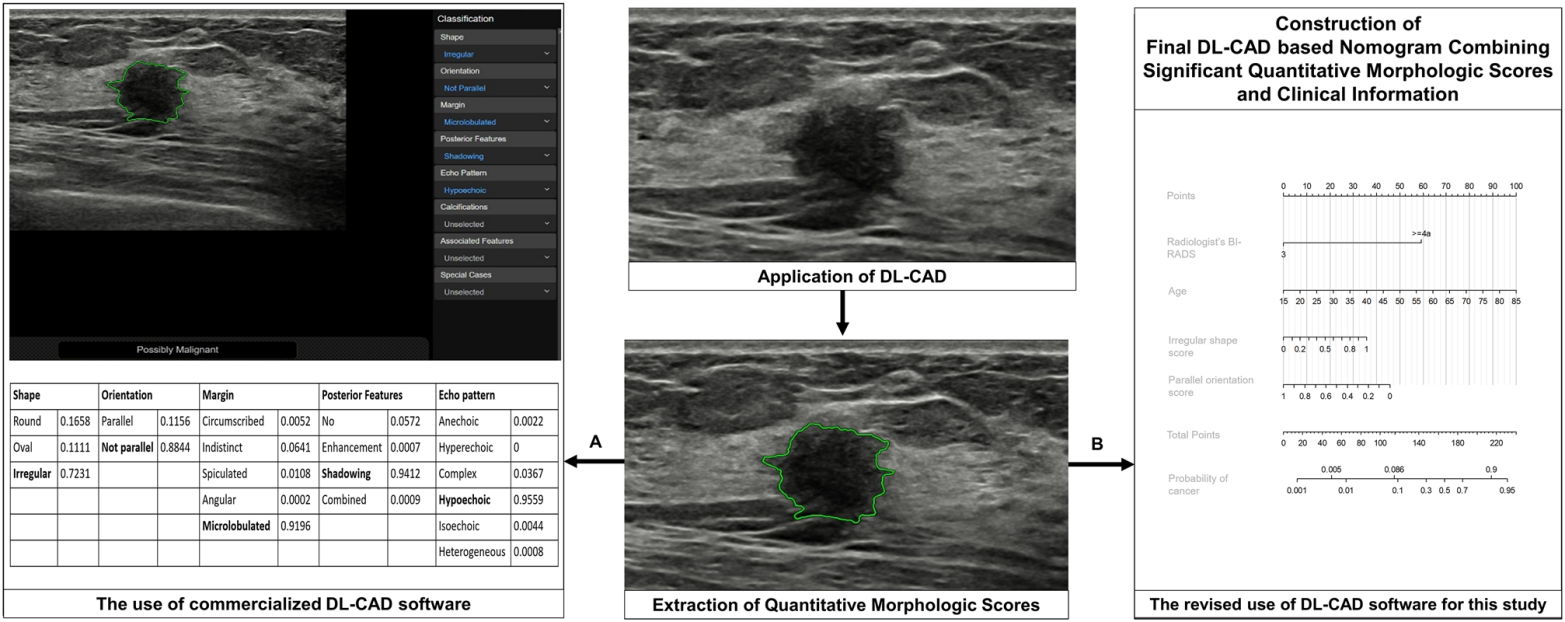
Application of deep learning based computer-aided diagnosis (DL-CAD) software in screening US-detected masses in (**a**) current commercialized mode and (**b**) our revised mode to build the diagnostic nomogram. A region of interest (ROI) (green line) was automatically drawn along the border of the screening US-detected mass in a 44-year-old woman, and the ROI based US features were analyzed by the DL-CAD software. Table in the left side shows quantitative scores obtained from DL-CAD software of the mass. The current commercial version of the DL-CAD software displays one descriptor with a maximum value for shape, orientation, margin, posterior echogenic features, and echo pattern: in this case, irregular shape, not parallel orientation, microlobulated margin, posterior shadowing, and hypoechoic echo pattern. Thus, these features and the final assessment of ‘possibly malignant’ were displayed on the monitor as an output (**a**). On the other hand, this study used the most significant quantitative morphologic scores extracted from the DL-CAD software and clinical information to build the diagnostic nomogram (**b**).

In this study, the quantitative morphologic scores obtained from the DL-CAD software, consisting of values between 0 and 1 for each BI-RADS lexicon descriptor, were used to develop a diagnostic model (Fig. 5) for the differential diagnosis of breast masses detected using screening US; this was because our unpublished preliminary test performed using the commercial DL-CAD software for the masses detected using screening US showed unsatisfactory diagnostic performance. The current commercial version of the DL-CAD software does not present these quantitative morphologic scores but displays one descriptor with a maximum value for the shape, orientation, margin, posterior echogenic features, and echo pattern. We believe that the current version involving the selection of one descriptor with a maximum value and dichotomized final assessment category might provide the omitted information with regard to our selected population with breast masses detected using screening US.

During the generation of the quantitative morphologic scores, deep learning technology was applied to build the classifier for the BI-RADS lexicon. A deep learning network consists of a series of multiple layers of simple components, many of which compute their own non-linear mappings between the input and output. Unlike conventional machine learning techniques, which use different algorithms and trained to learn about relationship between hand-crafted image features (i.e., edges, corners, or textures) and clinical outcomes, deep learning obtains the complex data structures in large high-dimensional datasets to determine the manner in which the internal parameters, called weights, of a deep neural network should be adjusted to learn the representations of data with multiple levels of abstraction. The weights are used to compute the representation of data in each layer based on the output of the previous layer, and complex functions with enough components of multiple levels of representation can be learned. For classification tasks, higher layers amplify the aspects of the input that are important for discrimination and suppress irrelevant variations^15^. All the weights of each layer are not generated by human experts but learned from a large amount of data via learning procedures. In the final layer, the inputs from the previous layer are transformed to probabilities that sum to one, representing the probability distributions of the final outputs. The final decision was made by combining the output of the US lexicon features with that of the other network for classifying the ROI images^29,30^.

### Data and statistical analysis

Along with the quantitative morphologic scores obtained from the DL-CAD software, the age of the patients, mass size based on the US, final BI-RADS assessment of the radiology report, and final pathological results were collected.

A comparison of the characteristics of the development and validation cohorts and those of the benign and malignant breast lesions in the development cohort was performed using the Chi-squared test for the categorical variables, the Wilcoxon rank sum test for the continuous variables except for age and size, and the independent t-test for age and size. Uni- and multivariable logistic regression analyses were used to identify the factors associated with malignancy in the development cohort. Variables with a *P* value < 0.1 under the univariable analysis were entered into the multivariable analysis with backward elimination. A nomogram was developed based on the multivariable logistic regression analysis. The diagnostic performance of the nomogram was evaluated in terms of discrimination and calibration. The discrimination was quantified using the area under the receiver operating characteristic (ROC) curve (AUC). The calibration was quantified using the calibration slope and intercept and visualized in the calibration curve. The internal validity of the nomogram was estimated via bootstrapping using 1000 bootstrap samples, whereas its external validity was estimated using the validation cohort. The hypothetical application of the nomogram was compared with the radiologists’ BI-RADS final assessment using the McNemar test in terms of the false positive rate, biopsy rate, and sensitivity. The cut-off of the nomogram was determined by maximizing the specificity without reducing the sensitivity to < 95% on the ROC curve. The cut-off of the radiologists’ BI-RADS final assessment was determined at category 4A. The false positive rate was defined as the number of benign breast lesions above the cut-off indicating suspicious for malignancy divided by the total number of benign lesions. The biopsy rate was defined as the number of breast lesions above the cut-off divided by the total number of breast lesions. The sensitivity was defined as the number of malignant lesions above the cut-off divided by the total number of malignant lesions. The SAS software (version 9.2; SAS Institute, Cary, NC) was used for the statistical analysis, and the R package (version 3.6.1) was used to build the nomogram.

## Supplementary information

Supplementary Table S1.
