# Supplementary material for: Deep learning-based computer-aided diagnosis in screening breast ultrasound to reduce false-positive diagnoses
Source: Sci Rep. 2021 Jan 11;11:395. doi: 10.1038/s41598-020-79880-0 (PMC7801712; doi:10.1038/s41598-020-79880-0)
Supplement: Supplementary file 1 — Supplementary Table S1. [file 41598_2020_79880_MOESM1_ESM.docx]

**Deep Learning-based Computer-Aided Diagnosis in Screening Breast Ultrasound to Reduce False-Positive Diagnoses**

Soo-Yeon Kim, M.D.^1^, Yunhee Choi, Ph.D.^2^, Eun-Kyung Kim, M.D.^3^, Boo-Kyung Han, M.D.^4^, Jung Hyun Yoon, M.D.^3^, Ji Soo Choi, M.D.^4^, Jung Min Chang, M.D.^1,*^

^1^Department of Radiology, Seoul National University Hospital, Seoul, Republic of Korea

^2^Medical Research Collaborating Center, Seoul National University Hospital, Seoul, Republic of Korea

^3^Department of Radiology and Research Institute of Radiological Science, Severance Hospital, Yonsei University College of Medicine, Seoul, Republic of Korea

^4^Department of Radiology and Center for Imaging Science, Samsung Medical Center, Sungkyunkwan University School of Medicine, Seoul, Republic of Korea

**Corresponding author**: Jung Min Chang, M.D. Ph.D.

Department of Radiology, Seoul National University College of Medicine,

101 Daehak-ro, Jongno-gu, Seoul, 03080, Republic of Korea

E-mail: imchangjm@gmail.com

Tel: 82-2-2072-0190

Fax: 82-2-747-7418

**Supplemental Table S1. Quantitative morphology scores of the development and validation cohorts from the deep learning based computer-aided diagnosis software**

| Characteristic | Descriptor | Development cohort  (n = 299) | Validation cohort  (n = 164) | *P* value |
| --- | --- | --- | --- | --- |
| Shape | Round | 0.01 (0, 0.05) | 0.05 (0.02, 0.13) | <.001 |
|  | Oval | 0.72 (0.26, 0.91) | 0.68 (0.36, 0.89) | .859 |
|  | Irregular | 0.17 (0.04, 0.68) | 0.15 (0.06, 0.47) | .701 |
| Orientation | Parallel | 0.99 (0.89, 0.99) | 0.99 (0.92, 0.99) | .545 |
|  | Not parallel | 0.01 (0, 0.11) | 0.01 (0, 0.08) | .545 |
| Margin | Circumscribed | 0.96 (0.19, 0.99) | 0.56 (0.08, 0.96) | <.001 |
|  | Indistinct | 0 (0, 0.07) | 0.09 (0.01, 0.52) | <.001 |
|  | Spiculated | 0 (0, 0) | 0 (0, 0) | <.001 |
|  | Angular | 0 (0 ,0 ,0 ,0.995) | 0 (0, 0) | <.001 |
|  | Microlobulated | 0.01 (0, 0.14) | 0.05 (0.01, 0.20) | <.001 |
| Posterior features | No | 0.45 (0.18, 0.73) | 0.79 (0.35, 0.97) | <.001 |
|  | Enhancement | 0.34 (0.07, 0.69) | 0.10 (0.01, 0.46) | <.001 |
|  | Shadowing | 0 (0, 0.01) | 0 (0, 0) | <.001 |
|  | Combined | 0 (0, 0.01) | 0 (0, 0) | <.001 |
| Echo pattern | Anechoic | 0 (0, 0) | 0 (0, 0.01) | <.001 |
|  | Hyperechoic | 0 (0, 0) | 0 (0, 0) | <.001 |
|  | Complex | 0 (0, 0) | 0 (0, 0.02) | <.001 |
|  | Hypoechoic | 0.85 (0.23, 0.98) | 0.37 (0.14, 0.68) | <.001 |
|  | Isoechoic | 0.05 (0, 0.43) | 0.28 (0.06, 0.74) | <.001 |
|  | Heterogeneous | 0 (0, 0) | 0.01 (0, 0.07) | <.001 |

Note:— Data represent the median values (25^th^ percentile, 75^th^ percentile).
